# Programmed death ligand 1 intracellular interactions with STAT3 and focal adhesion protein Paxillin facilitate lymphatic endothelial cell remodeling

**DOI:** 10.1016/j.jbc.2022.102694

**Published:** 2022-11-12

**Authors:** Johnathon B. Schafer, Erin D. Lucas, Monika Dzieciatkowska, Tadg Forward, Beth A. Jirón Tamburini

**Affiliations:** 1 Department of Medicine, Division of Gastroenterology and Hepatology, University of Colorado School of Medicine, Aurora, Colorado, USA; 2 Molecular Biology Graduate Program, University of Colorado School of Medicine, Aurora, Colorado, USA; 3 Immunology Graduate Program, University of Colorado School of Medicine, Aurora, Colorado, USA; 4 Department of Biochemistry, University of Colorado School of Medicine, Aurora, Colorado, USA; 5 Department of Immunology and Microbiology, University of Colorado School of Medicine, Aurora, Colorado, USA

**Keywords:** Lymph node, Immunology, Lymphatic endothelial cell, PD-L1, Reverse-signaling, Cellular movement, F-actin, Mass spectrometry, STAT3, Paxillin, DC, dendritic cell, FBS, fetal bovine serum, LEC, Lymphatic endothelial cell, LN, lymph node, MS, mass spectrometry

## Abstract

Lymphatic endothelial cells (LECs) comprise lymphatic capillaries and vessels that guide immune cells to lymph nodes (LNs) and form the subcapsular sinus and cortical and medullary lymphatic structures of the LN. During an active immune response, the lymphatics remodel to accommodate the influx of immune cells from the tissue, but factors involved in remodeling are unclear. Here, we determined that a TSS motif within the cytoplasmic domain of programmed death ligand 1 (PD-L1), expressed by LECs in the LN, participates in lymphatic remodeling. Mutation of the TSS motif to AAA does not affect surface expression of PD-L1, but instead causes defects in LN cortical and medullary lymphatic organization following immunostimulant, Poly I:C, administration *in vivo*. Supporting this observation, *in vitro* treatment of the LEC cell line, SVEC4-10, with cytokines TNFα and IFNα significantly impeded SVEC4-10 movement in the presence of the TSS-AAA cytoplasmic mutation. The cellular movement defects coincided with reduced F-actin polymerization, consistent with differences previously found in dendritic cells. Here, in addition to loss of actin polymerization, we define STAT3 and Paxillin as important PD-L1 binding partners. STAT3 and Paxillin were previously demonstrated to be important at focal adhesions for cellular motility. We further demonstrate the PD-L1 TSS-AAA motif mutation reduced the amount of pSTAT3 and Paxillin bound to PD-L1 both before and after exposure to TNFα and IFNα. Together, these findings highlight PD-L1 as an important component of a membrane complex that is involved in cellular motility, which leads to defects in lymphatic organization.

Lymphatic endothelial cells (LECs) comprise the lymphatic capillaries and vessels, which are essential for antigen and immune cell trafficking from the peripheral tissue to draining lymph nodes (LNs) (1, 2). The LECs make up the lymphatic structures in the LN including the lymphatic sinus, cortical lymphatics, and medullary lymphatics (3, 4, 5). These structures are found not only in different locations of the LN but have unique functions that rely on distinct transcriptional profiles (3, 4, 5, 6). During an active immune response, lymphatic capillaries found in the peripheral tissue recruit CCR7 expressing immune cells to the draining LN *via* the chemokine CCL21, the ligand of CCR7 (7). LECs also upregulate cellular adhesion molecules Intercellular adhesion molecule-1 (ICAM1) and vascular cell adhesion molecule-1 (VCAM1) to facilitate LEC–immune cell interactions (5, 8, 9). Secretion of inflammatory chemokines, such as tumor necrosis factor-α (TNFα) and type 1 interferon (IFN), result in the upregulation of these cellular adhesion molecules and chemokines in order to facilitate LEC-dendritic cell (DC) interactions (9, 10, 11). Early on in the immune response type 1 IFN production also results in the upregulation of Programmed Death Ligand 1 (PD-L1) on the LN LECs (11). Type 1 IFN suppresses LEC division early during the immune response and cooperates with PD-L1 to regulate LEC division throughout the immune response (11, 12). Following the brief inhibition of growth by type I IFN and PD-L1, the cortical LN lymphatics expand to accommodate the influx immune cells (2, 11). The regulation of this LEC expansion is in response to production of vascular endothelial growth factor-A (VEGF-A) from B-cells or VEGF-A and VEGF-C from fibroblastic reticular cells, DCs, and macrophages (12, 13, 14). Activated T-cells also trigger LN lymphatic expansion, but the mechanism of this expansion is unknown (2). However, secretion of IFNγ by T-cells at the conclusion of the immune response is necessary for lymphatic apoptosis and LN contraction to return the LN into the homeostatic state (15).

The expansion of the LN lymphatics, important for immune cell infiltration to the LN during an active immune response, has largely been studied on a system level. Where inflammatory cytokines that regulate the function and expansion (TNFα, IFNα, PD-L1, LTα, VEGF-A, VEGF-C, VEGF-D) coordinate the reorganization and proliferation of lymphatic structures in the LN (11, 12, 16, 17, 18, 19). In an *in vitro* culture system, LECs exposed to TNFα upregulated adhesion proteins and induced actin polymerization, resulting in longer continuous F-actin filaments (20). Also *in vitro*, TNFα negatively regulated LEC proliferation and facilitated organization into capillary structures (21). In the absence of inflammatory cytokines, LEC migration and proliferation in response to VEGF occurred concurrently with F-actin fiber formation (22, 23). While little has been described regarding the molecular mechanisms required for the movement and reorganization of the lymphatics of the LN, the studies aforementioned suggest that F-actin fiber formation following TNFα, and likely type 1 IFN, facilitate lymphatic remodeling.

In the naïve mouse, LN LECs have variable expression of PD-L1. PD-L1 is expressed at relatively high levels on floor LECs, Marco-LECs, and tzLECs (3, 4, 5, 6, 11, 24). This expression of PD-L1 under normal conditions is important in maintaining tolerance to peripheral tissue antigens and preventing aberrant lymphatic proliferation (11, 24). Following a type 1 IFN inducing stimulus, all LECs upregulate PD-L1 (11). The resulting upregulation of PD-L1 by LECs impedes cell division and improves LEC survival, a function which was lost in the absence of PD-L1 (11). Thus, PD-L1 significantly affects LEC function; however, to our knowledge, there is no evidence for how PD-L1 affects intracellular signaling in LECs to manipulate LEC function. PD-L1 reverse signaling, however, has been described in cancer cells (25, 26, 27, 28, 29, 30, 31, 32). Intriguingly, in cancer cells, PD-L1 reverse signaling caused cellular migration and increased survival following treatment with type 1 IFN, chemotherapeutics, or radiation (25, 26, 28, 30, 31, 32). The protection from type 1 IFN was suggested to be a result of increased pSTAT3 activation in the absence of PD-L1, resulting in Caspase3/7 activation (28). Another report demonstrated defective migration in the absence of PD-L1, caused by interactions between PD-L1 and H-Ras, which led to downstream MEK and ERK phosphorylation (31).

In cancer cells, two intracellular domains were identified to regulate PD-L1 in response to type 1 IFN, residues 264 to 273 and residues 275 to 281 (28). An additional study demonstrated that residues 270 to 279 of PD-L1 were required to interact with and stabilize mRNA (32). This dataset demonstrated that PD-L1 could act to regulate DNA damage repair enzymes *via* the mRNA-PD-L1 interaction (32). Evaluation of mRNA molecules shown to bind to PD-L1 based on their data did not provide clues regarding why or how PD-L1 may regulate cellular movement. In our previous report (1), we demonstrated that a specific cytoplasmic motif, threonine-serine-serine (TSS), is responsible for at least some of the defined intracellular signaling by PD-L1. The function of this motif was demonstrated by mutation of amino acids 277 to 279 TSS to alanine-alanine-alanine (AAA) (1). In DCs, the three amino acid mutation in the cytoplasmic domain of PD-L1 caused defective chemokine receptor signaling, loss of ERK phosphorylation, and decreased actin polymerization (1). Loss of the TSS motif in PD-L1 led to defective chemotaxis of DCs but did not alter surface expression of PD-L1 (1). While PD-L1 expression by LECs and consequences of loss of PD-L1 in LECs has been demonstrated, whether the TSS motif functions similar in LECs as DCs is yet unknown.

Here, we demonstrate that loss of three residues within the cytoplasmic domain of PD-L1 significantly impairs LN lymphatic reorganization following poly I:C injection into the footpad of mice. We produced a stable LEC line with constitutive expression of either WT *Pdl1* or *Pdl1* with the TSS-AAA mutation in the cytoplasmic domain (*Pdl1*^*CyMt*^*).* We observed a similar growth phenotype and expression of PD-L1 in these cells at steady state. Upon stimulation with either type 1 IFN or TNF alpha, we show a significant defect in actin polymerization and cellular movement across a wound. These phenotypic changes appear to be a result of defective intracellular interactions between PD-L1, pSTAT3, and paxillin. Interestingly, pSTAT3 and paxillin were previously reported to form a complex at focal adhesions, which are important for regulating actin polymerization required for cellular movement (33, 34). Together, our data clearly demonstrate that the intracellular domain of PD-L1 contributes to membrane protein interactions that regulate motility and that these interactions are critical for lymphatic remodeling.

## Results

### PD-L1 facilitates lymphatic reorganization following poly I:C

**Figure 1:**
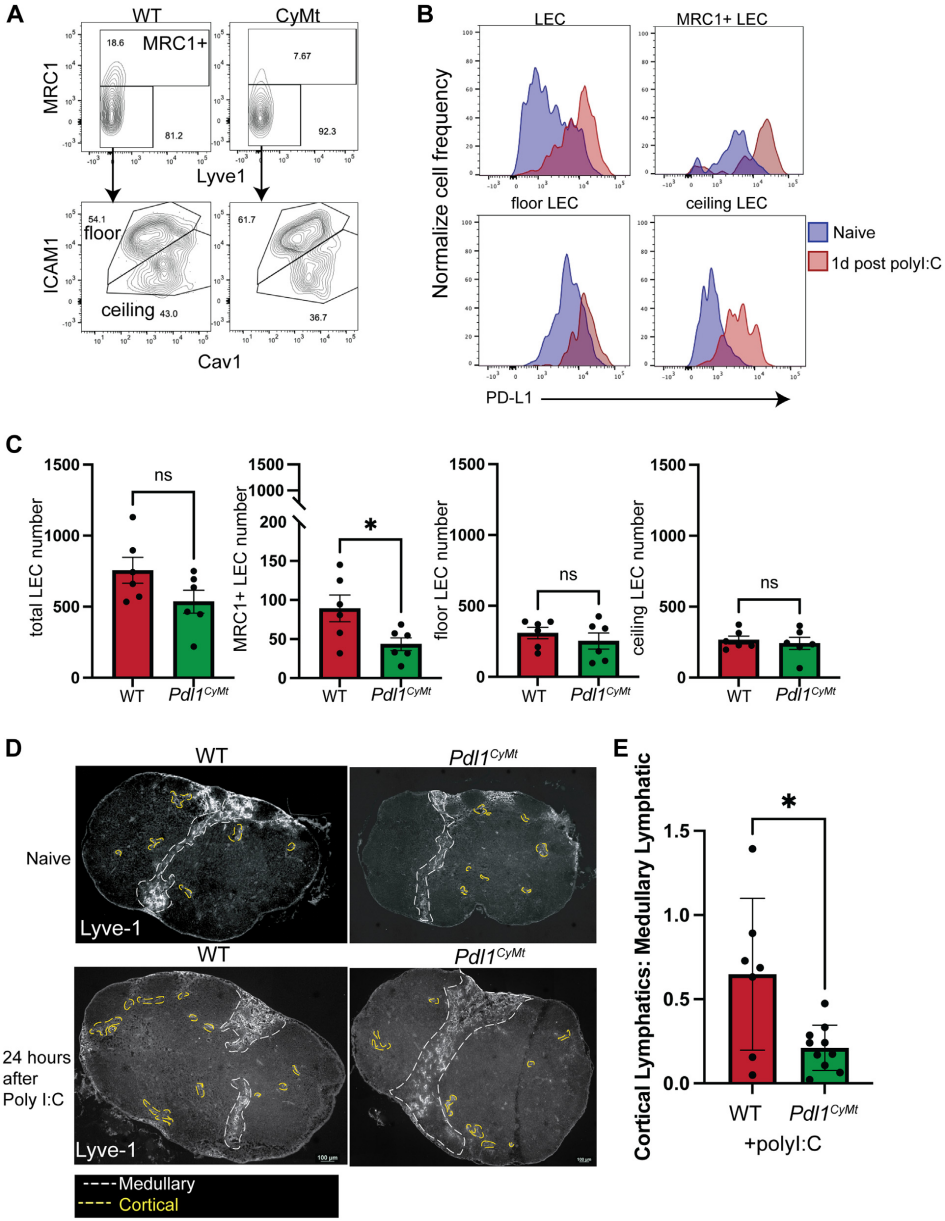
**Mutation of the cytoplasmic domain of PD-L1 reduces MRC1+ LECs and alters lymphatic reorganization following poly I:C.***A*, mice were injected with 5 μg poly I:C in 50 μl PBS in the footpad and flank. Mice were then sacrificed 24 h later and popliteal as well as inguinal lymph nodes (LNs) were minced, digested, and stained for flow cytometry analysis of different LEC subsets. LECs were gated on CD45-, PDPN+, CD31+. Subsets were defined as MRC1+ LECs, MRC1-LECs were either ICAM1^hi^ CAV1^low^ (floor LECs) or ICAM1^lo^ CAV1^hi^ (ceiling). *B*, PD-L1 expression was determined before and after poly I:C. *C*, number of LECs in subsets was compared between WT and *Pdl1*^*CyMt*^. *D*, LNs were fixed with formalin, embedded in paraffin wax, and then sectioned in 7 μm slices onto glass slides. Sections stained for LYVE-1 to visualize lymphatic endothelial cells (*white)*. Morphological areas were determined as either medullary (*white*) or cortical (*yellow*) lymphatics. *E*, the ratio of lymphatic area of the cortical lymphatics compared to the medullary lymphatics was quantified in LNs of mice 24 h after poly I:C. Data show pooled quantification from two experiments. Students *t* test was used to compare groups∗=*p* < 0.05. LEC, Lymphatic endothelial cell.

We had previously identified a cytoplasmic motif region within murine PD-L1 that contributed to DC chemotaxis (1) and demonstrated that PD-L1 was important for LEC survival (11). As DC and LEC movement and survival are important components of LN organization and responsiveness, we asked if there were differences in the LNs of WT or *Pdl1*^*CyMt*^ mice, in which the cytoplasmic TSS motif is mutated to AAA (Supplemental Fig. 1*A*). We first evaluated LEC subsets by flow cytometry based on transcriptional signature as defined in (6) in both WT and *Pdl1*^*CyMt*^ mice. We identified LECs as CD45-negative, Podoplanin (PDPN)-positive, CD31-positive cells (Supplemental Fig. 1*B*). We further identified cortical/medullary LECs based on Lyve-1 and Mannose Receptor C type 1 (MRC-1) expression and ceiling and floor LECs based on Intercellular Adhesion Molecule-1 (ICAM1) and Caveolin-1 (CAV1) expression before and after poly I:C (Fig. 1*A*). We saw that poly I:C caused the upregulation of PD-L1 on all LEC subsets (Fig. 1*B*) and that there was no difference in upregulation of PD-L1 between WT and the *Pdl1*^*CyMt*^ LECs(Supplemental Fig. 1, *C* and *D*). We next compared the number of LECs in each subset and found no differences in any subset except the MRC1-positive LECs, which were fewer in frequency and number in the *Pdl1*^*CyMt*^ (Fig. 1, *A* and *C*). To evaluate lymphatic organization in the LNs of WT and *Pdl1*^*CyMt*^ mice before and after poly I:C, we performed immunostaining for Lymphatic Vessel Hyaluronan Receptor 1 (Lyve-1) (Fig. 1*D*). Each LN was sectioned and stained for Lyve-1 (35) (Fig. 1, *D* and *E*). Sections revealed that the lymphatics of naïve LNs look similar between WT and *Pdl1*^*CyMt*^ mice. After poly I:C injection, we found that in WT mice there was reorganization of both the medullary and cortical LECs as previously demonstrated (11, 13, 36). Cortical lymphatics were delineated as lymphatics not connected to the subcapsular sinus (*yellow* dashed lines). Medullary lymphatics were delineated as lymphatics anatomically located in the medulla of the LN and connected to the subcapsular sinus (*white* dashed lines) (37). The draining LN lymphatic vasculature of *Pdl1*^*CyMt*^ mice given poly I:C was largely comprised of lymphatics within the medullary area with minimal occupancy of lymphatics in the cortical area (Fig. 1*D*). Upon quantification, the *Pdl1*^*CyMt*^ cortical lymphatics were decreased relative to the medullary lymphatics in the LNs after poly I:C injection into the mouse footpad or flank (Fig. 1*E*). These findings demonstrate that the TSS region within the cytoplasmic domain of PD-L1 is important for regulating lymphatic organization during immune activation with poly I:C.

### Stable transduction and constitutive expression of *Pdl1* and *Pdl1*^*CyMt*^ does not impair growth

In order to determine the contribution of the TSS motif of PD-L1 to reverse signaling in the LECs, we transduced an SVEC4-10 cell line, a cell line that has been previously described to be of lymphatic origin (38). SVEC4-10 cells were transduced with pBABE-GFP vectors containing either GFP alone (empty vector), WT *Pdl1* tagged with GFP (*Pdl1*), or *Pdl1*^*CyMt*^ tagged with GFP (*Pdl1*^*CyMt*^) (Supplemental Fig. 2*A*). Upon stable transduction with the lentiviral vector containing WT or mutant PD-L1 (*Pdl1*^*CyMt*^), SVEC4-10 cells growth rate (Supplemental Fig. 2*B*) and surface expression of PD-L1 (Supplemental Fig. 2, *C* and *D*) were measured over 8 days (Supplemental Fig. 2, *B* and *D*). Similar to LECs from mice that harbor the *Pdl1*^*CyMt*^ mutation (Supplemental Fig. 1*D*), expression of PD-L1 was equivalent between WT and *Pdl1*^*CyMt*^ transduced cells and there was no difference in growth rate (Supplemental Fig. 2, *B*–*D*). SVECs normally have extremely low levels of PD-L1 but upregulate PD-L1 following treatment with IFNα, TNFα, or both IFNα and TNFα (Supplemental Fig. 2, *E* and *F*). However, the PD-L1-transduced cells constitutively express high levels of PD-L1 compared to endogenous PD-L1 regardless of treatment (Supplemental Fig. 2*F*).

### PD-L1 cytoplasmic mutation reduces cellular movement in the presence of TNF**α** and IFN**α**

**Figure 2:**
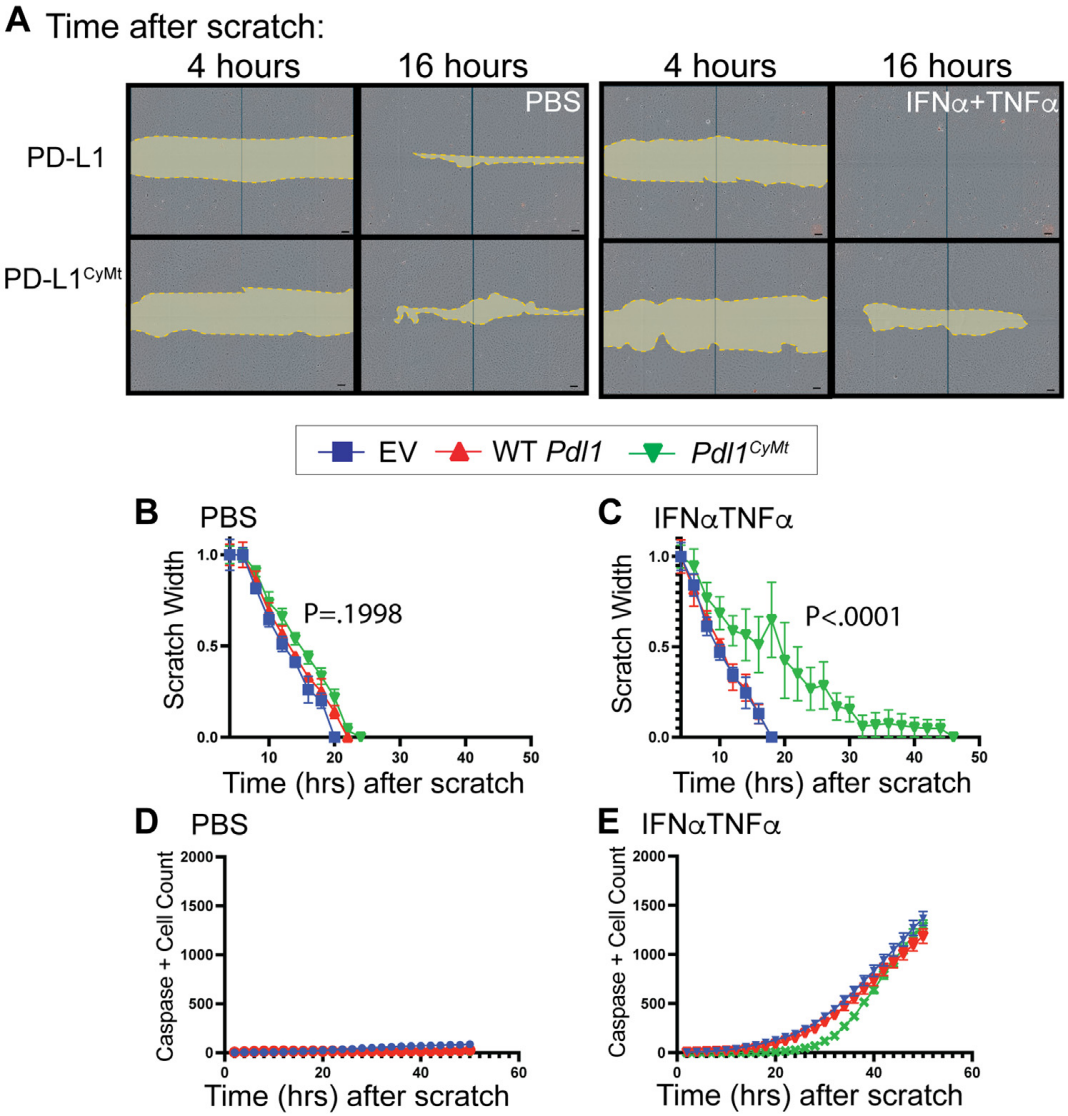
**Mutation of PD-L1 significantly impairs SVEC movement but not viability.***A*, SVEC 4-10 cells containing EV, WT *Pdl1*, or *Pdl1*^*CyMt*^ vectors were plated in five wells per group per treatment, of an Image-Lock 96-well plate, at 1.5e^4^ cells/well. Cells were allowed to grow to confluence ∼36 h. Confluent cells were scratched with a Sartorius Woundmaker. Immediately after scratch, media was changed to serum-free MEM media containing either PBS, TNFα (100 ng/ml), IFNα (500 U/ml), or both TNFα (100 ng/ml) and IFNα (500 U/ml). Cells were imaged every 2 h using an IncuCyte. *A*, representative images and representative graphs are shown from three independent experiments, 5 to 7 wells each. *B* and *C*, quantification of scratch width over time after (*B*) PBS and (*C*) IFNα and TNFα at concentrations indicated before. Graph is of representative experiment. Assay was repeated three independent times with similar results. *p*-value for difference in slope of WT *Pdl1 versus Pdl1*^*CyMt*^ best fit line shown. *D* and *E*, number of active Caspase 3 reagent positive cells to determine the number of apoptotic cells per image over time. No significant differences were found in caspase three between *Pdl1* and *Pdl1*^*CyMt*^. The scale bars represent 100 μm.

After confirming that the proliferation and surface expression was unaffected by overexpression of *Pdl1*^*CyMt*^ compared to WT *Pdl1*, we tested the ability of these cells to reorganize following disruption of a monolayer (Fig. 2*A*). We observed that after scratching an SVEC monolayer, transduced SVECs were capable of quickly closing the wound regardless of which *Pdl1* construct they contained (Fig. 2, *A* and *B*). These findings align with our *in vivo* observations where the *Pdl1*^*CyMt*^ mutation does not alter LEC structures at homeostasis (Fig. 1*D*). To determine if the cytokines TNFα and IFNα impacted wound closure, we treated transduced SVECs with either TNFα, IFNα or a combination of the two, to mimic the cytokine production by cells *in vivo* following poly I:C. Cells expressing *Pdl1*^*CyMt*^ exhibited a significant delay in wound closure with type 1 IFN (Supplemental Fig. 3, *A* and *B*), TNFα (Supplemental Fig. 3, *A* and *C*), and an even more pronounced defect with a combination of type 1 IFN and TNFα (Fig. 2, *A* and *C*). The pattern of cell movement was observed to be different between cells expressing WT *Pdl1* and *Pdl1*^*CyMt*^*.* The increased number of cells leaving the cell-cell contacts of the scratch edge and migrating independently to the center of the scratch in the *Pdl1*^*CyMt*^ cells suggests defects in coordinated cellular movement (Supplemental Fig. 3, *D* and *E*). During this time period, there was no difference in the number of apoptotic cells between WT *Pdl1* and *Pdl1*^*CyMt*^ (Fig. 2, *D* and *E* and Supplemental Fig. 3, *F* and *G*) and no difference in cell growth between groups over a 5 day period (Supplemental Fig. 3, *H*–*K*). This suggested that PD-L1 was required for LEC remodeling of the scratch when signaling from the inflammatory cytokines, type I IFN and TNFα was active, but not during homeostasis.

### Cells expressing *Pdl1*^*CyMt*^ are defective in F-actin polymerization

**Figure 3:**
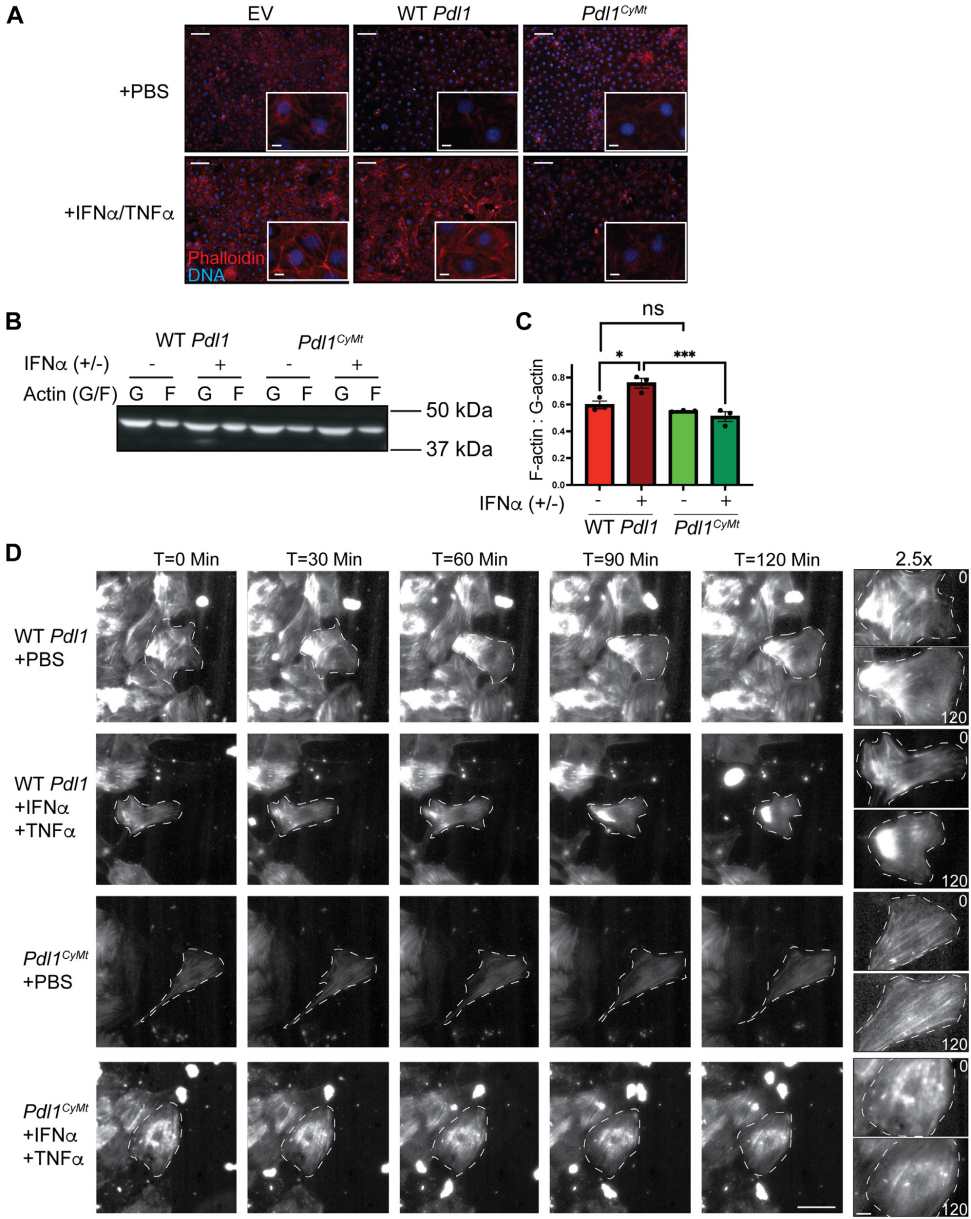
**Actin polymerization in *Pdl1***^***CyMt***^**expressing SVEC cells is impaired.***A*, cells grown on collagen-coated coverslips were treated with either PBS or TNFα (100 ng/ml) and IFNα (500 U/ml), then fixed and stained with Phalloidin to visualize F-actin fluorescence (*red*) and DAPI (*blue*). *B*, cells grown in 6-well plates were treated with IFNα (500 U/ml) and lysed. F-actin was pelleted from G-actin using centrifugation and fractions were run on an SDS gel, transferred to a PVDF membrane, and probed for actin *via* Western blot. *C*, F-actin/G-actin ratio was determined based on band intensity from Western blot using three independent experiments. *D*, in conditions identical to the scratch assay, cells were stained with SiR-actin live cell probe. Cells were imaged every 10 min over time after treatment. Frames from the first 2 h of imaging were selected every 30 min to show changes in cell morphology and actin. Dashed lines indicate cell borders and shape. 2.5× zoomed in images of cells with dashed lines are shown from times 0 and 120 min. Statistics were performed using a one-way ANOVA on 3 independent experiments. The scale bar in (*A*) represents 100 μm and 10 μm for inset. The scale bar in (*D*) represents 50 μm and 10 μm in zoomed in images of F-actin. ∗=*p* < 0.05, ∗∗=*p* < 0.01, ∗∗∗=*p* < 0.001. DAPI, 4′,6-diamidino-2-phenylindole; PVDF, polyvinylidene difluoride.

Based on our findings in Fig. 1 that *Pdl1*^*CyMt*^ LN lymphatics are improperly remodeled after poly I:C and that *Pdl1*^*CyMt*^ SVEC cells are defective in wound closure after type 1 IFN and TNFα in Fig. 2, we next asked about F-actin levels in SVEC cells after stimulation with TNFα and/or IFNα. To do this, we stained SVEC 4 to 10 cells with stable expression of *Pdl1*, *Pdl1*^*CyMt*^, or empty vector with phalloidin conjugated to a fluorophore (F-actin) (Fig. 3*A*). We noticed that while there was no difference in the intensity of F-actin staining or organization between the groups treated with vehicle (PBS), that there was an increase in the intensity and difference in the apparent organization of F-actin upon treatment with IFNα and TNFα with WT *Pdl1* that was absent in the cells expressing *Pdl1*^*CyMt*^ (Fig. 3*A*). In order to quantify the cytoskeletal differences in actin, we evaluated the F-actin/G-actin ratio. We found that WT *Pdl1* and *Pdl1*^*CyM*^^*t*^ transduced SVEC cells have similar levels of F-actin/G-actin without stimulation (Fig. 3, *B* and *C*). Similar to our staining with F-Actin (Fig. 3*A*), we found significant differences in F-actin/G-actin ratio following type 1 IFN. Cells expressing WT *Pdl1* had increased levels of F-actin that were absent in the cells expressing *Pdl1*^*CyMt*^ (Fig. 3*B*)*.* Furthermore, upon evaluation of the ratio of F-actin to G-actin, we found a significant decrease in the ratio in the *Pdl1*^*CyMt*^ cells compared to WT *Pdl1* expressing cells due to the lack of F-actin polymerization in the *Pdl1*^*CyMt*^ cells rather than loss of F-actin (Fig. 3*C*). To demonstrate differences in actin reorganization, we imaged transduced WT *Pdl1* and *Pdl1*^*CyMt*^ cells with a live-cell F-actin probe after wounding and treatment (as in Fig. 2*A*) with vehicle (Movies 1 and 2), with IFNα (Movies 3 and 4), with TNFα (Movies 5 and 6), or after treatment with both IFNα and TNFα (Movies 7 and 8). In the WT *Pdl1* and *Pdl1*^*CyMt*^ cells treated with vehicle, we observed actin reorganization over time as the cells migrated into the scratch (Fig. 3*D* and Movies 1 and 2). However, in the *Pdl1*^*CyMt*^ compared to WT *Pdl1,* with both IFNα and TNFα, there was both reduced movement and less F-actin reorganization (Fig. 3*D* and Movies 1,2,7,8). These findings demonstrate a significant impairment in actin polymerization and reorganization in the presence of cytokine stimulation, which is consistent with ineffective lymphatic remodeling *in vivo*.

### PD-L1 intracellular interactions

**Figure 4:**
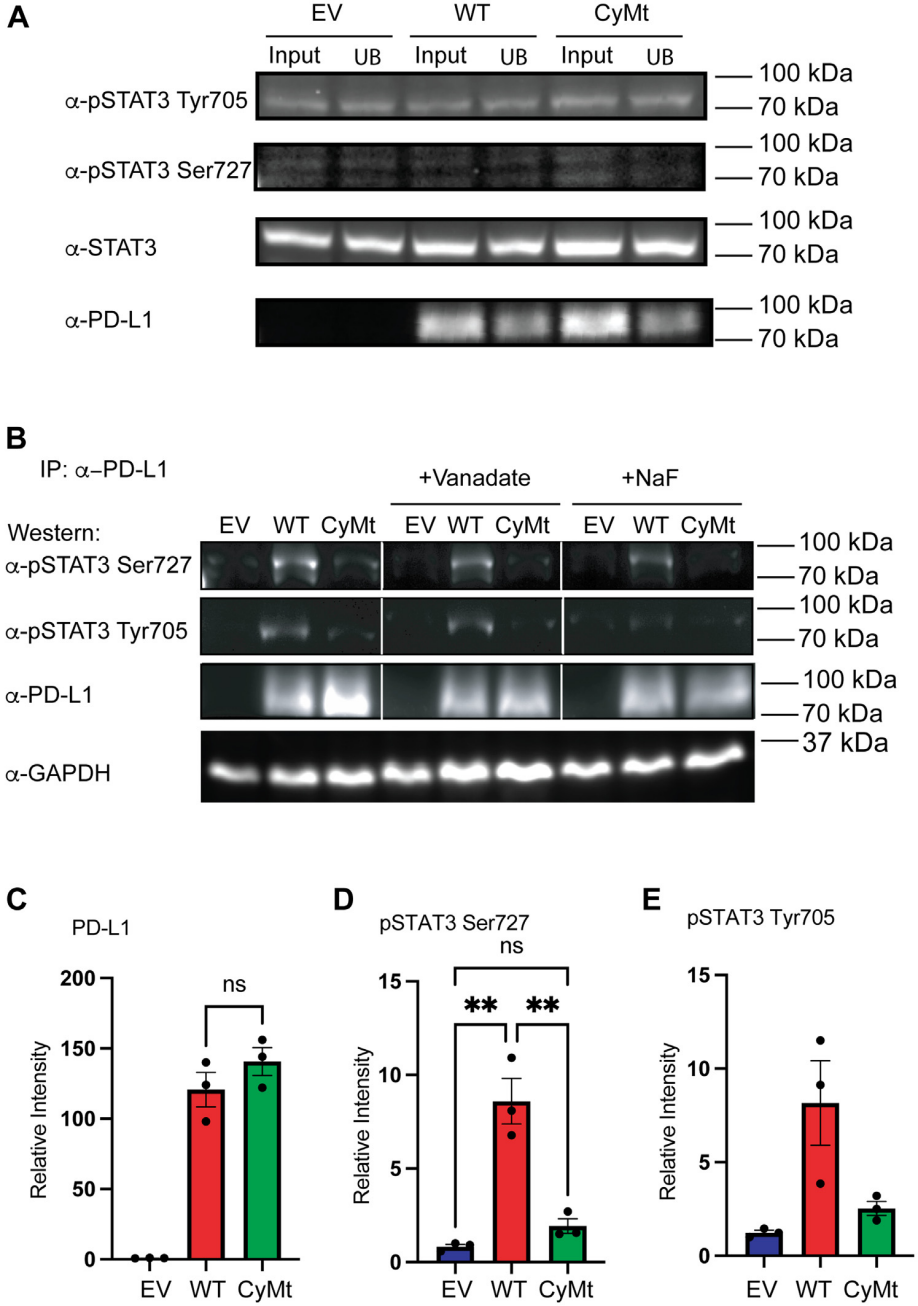
**Immunoprecipitation of PD-L1 followed by probing for STAT3 phosphorylation sites in the presence of phosphatase inhibitors.***A*, input and unbound samples for PD-L1 immunoprecipitation samples from WT *Pdl1* or *Pdl1*^*CyMt*^ cells. *B*, western blots for pSTAT3 Ser727, pSTAT3 Y705, and PD-L1 following immunoprecipitation for PD-L1 from WT *Pdl1* or *Pdl1*^*CyMt*^ cells. *C*–*E*, quantification of PD-L1, pSTAT3 Ser727, or pSTAT3 Tyr705. One-way ANOVA on 3 combined experiments performed. ∗∗=*p* < 0.01.

To begin to understand what protein–protein interactions with PD-L1 may contribute to differences in actin polymerization, we performed mass spectrometry (MS) on cellular lysates after an immunoprecipitation of PD-L1. SVECs overexpressing either PD-L1 or PD-L1^CyMt^ were immunoprecipitated with or without sodium vanadate (a phosphatase inhibitor) (Supplemental Fig. 4). Immunoprecipitated proteins were assessed by MS to determine potential interactions that relied on the TSS domain of PD-L1. Several proteins were identified by MS (Table 1 and Supplemetal Table 1). Among those proteins that differed between WT PD-L1 and PD-L1^CyMt^ were Paxillin, a protein involved in cellular focal adhesions (39) and STAT3, a protein shown to bind to Paxillin (34) and have increased tyrosine phosphorylation in the absence of PD-L1 or with the mutation (1, 28) (Table 1). As STAT3 is regulated by phosphorylation, we next asked if, in our vanadate-treated samples, there were differences in the phosphorylation state of either PD-L1 or proteins bound to PD-L1. Therefore, lysates were enriched for phosphorylated proteins and isolated for MS. Only 29 phosphorylated proteins were identified and only five were found in the WT sample but not the PD-L1^CyMt^ sample, including Krt5, Stat3, Arghap35, Ifih1, and Heatr5b (Table 2). Interestingly, the STAT3 peptide isolated using MS contained Serine 727 (Table 3), a modification important in both STAT3 regulation of mitochondrial respiration (40, 41, 42, 43, 44, 45) and regulation of STAT3 Tyr705 activation of transcription (46, 47). To confirm differences in binding of phosphorylated forms of STAT3, we next performed western blot analysis of both sodium vanadate (inhibitor of threonine phosphatases) and sodium fluoride (inhibitor of serine phosphatases) treated cells following PD-L1 immunoprecipitation. There was not a difference in the total levels put into the immunoprecipitation of either PD-L1, pSTAT3 Ser727, or Tyr705 between WT PD-L1 and PD-L1^CyMt^ samples (Fig. 4*A*). Evaluation of the immunoprecipitated product revealed STAT3 was pulled down with PD-L1 (Table 1, Fig. 4*B*) and that of the pulled down fraction STAT3 was phosphorylated on both Ser727 and Tyr705 regardless of the phosphatase inhibitor used (Table 2, Fig. 4*B*). Quantification of these data confirm that the amount of PD-L1 pulled down was not significantly different between WT and *Pdl1*^*CyMt*^ expressing cells (Fig. 4*C*). The amount of phosphorylated pSer727 STAT3 bound to PD-L1 was significantly increased, even in the absence of phosphatase inhibitors, and this interaction was significantly impaired with PD-L1^CyMt^ (Fig. 4*D*). Similar trends were seen when evaluating PD-L1 interactions with pTyr705 STAT3 (Fig. 4*E*). Changes in the interactions with pSTAT3 could be caused by increased total levels of phosphorylated Tyr705 STAT3 following stimulation, which has been previously demonstrated in both DCs (1) and cancer cells (28) after type 1 IFN. However, when we asked if the STAT3 or pSTAT3 interaction was changed in the presence of IFNα, TNFα, or both, we again found a significant reduction in the interaction between pSTAT3 Ser727 and PD-L1 but not pSTAT3 Tyr705 and PD-L1 (Supplemental Fig. 5, *A* and *B*).

**Table 1:** Mass spectrometry analysis following pull down of GFP-tagged WT PD-L1 and PD-L1^CyMt^

Protein name	Alternate ID	Molecular weight	Number of samples identified in WT Pdl1 after IP	Number of samples identified in Pdl1^CyMt^ after IP
RuvB Like AAA ATPase 2	Ruvbl2	51 kDa	5	0
Salt Inducible Kinase 2	Sik2	104 kDa	5	0
Prolyl 4-Hydroxylase Subunit Alpha 1	P4ha1	61 kDa	4	0
Glutathione Peroxidase 7	Gpx7	21 kDa	4	0
Paxillin	Pxn	64 kDa	4	0
Thyroid Hormone Receptor Alpha	Thra	55 kDa	3	0
DnaJ Heat Shock Protein Family (Hsp40) Member C7	Dnajc7	56 kDa	4	0
Perilipin 2	Plin2	47 kDa	3	0
Solute Carrier Family 25 Member 3	Slc25a3	40 kDa	3	0
T-cell-specific guanine nucleotide triphosphate-binding protein 1	Tgtp1	47 kDa	3	0
NOP58 Ribonucleoprotein	Nop58	60 kDa	3	0
Glutamine Rich 1	Qrich1	87 kDa	3	0
OTU Deubiquitinase 7B	Otud7b	92 kDa	3	0
2′-5′-Oligoadenylate Synthetase 2	Oas2	85 kDa	3	0
Obscurin Like Cytoskeletal Adaptor 1	Obsl1	198 kDa	3	0
HDGF Like 2	Hdgfl2	74 kDa	3	0
TBC1 Domain Family Member 23	Tbc1d23	76 kDa	3	0
Rho Gtpase Activating protein 29	Arhgap29	142 kDa	3	0
Adenosylhomocysteinase Like 1	Ahcyl1	59 kDa	3	0
Signal transducer and activator of transcription 3	STAT3	88 kDa	6	6
Programmed cell death 1 ligand 1 (Cd274)	PD-L1	33 kDa	6	6
Rho GTPase Activating Protein 35	ARHGAP35	170 kDa	6	6

Mass spectrometry analysis of proteins pulled down by immunoprecipitation (IP) with GFP-tagged WT *Pdl1* or *Pdl1*^*CyMt*^ samples identified proteins bound to both as well as other proteins identified in only WT *Pdl1* or *Pdl1*^*CyMt*^ samples. Table is curated showing 19 of the most enriched proteins identified as possible preferentially binding to WT PD-L1 as well as other proteins of interest.

**Table 2:** Phosphorylated proteins coimmunoprecipitated

Protein name	Alternative ID	Molecular weight	Number identified in WT	Number identified in mut
Keratin 5	Krt5	62 kDa	1	0
Signal Transducer and Activator of Transcription 3	Stat3	88 kDa	1	0
MDA5	Ifih1	116 kDa	1	0
Rho GTPase Activating Protein 35	Arhgap35	170 kDa	1	0
Heat repeat containing 5b	Heatr5b	224 kDa	1	0
ATP Binding Cassette Subfamily F Member 1	Abcf1	95 kDa	0	1
Cordon-bleu Protein Like-1	Cobll1	137 kDa	0	1
COPI Coat Complex Subunit Alpha	Copa	138 kDa	0	1
Rho Guanin nucleotide exchange factor 40	Arhgef40	165 kDa	0	1
N-Alpha-Acetyltransferase 15, NatA Auxiliary Subunit	Naa15	101 kDa	0	1
Drebin 1	Dbn1	77 kDa	0	1
Striatin	Strn	86 kDa	0	1
Scaffold Attachment Factor B2	Safb2	112 kDa	0	1
General Transcription Factor IIi	Gtf2i	112 kDa	0	1
Pleckstrin Homology Like Domain Family B Member 2	Phldb2	141 kDa	0	1

PD-L1-GFP was immunoprecipitated from lysates of SVEC4-10 cells expressing either WT *Pd-l1* or *Pd-l1*^*CyMt*^ following treatment with sodium vanadate. Samples were enriched for phosphorylated peptides and analyzed by mass spectrometry. Phosphorylated proteins identified in samples are shown.

**Table 3:** Phosphopeptide analysis

Specific peptides identified by mass spectrometry following phosphopeptide enrichment shown from Table 2. Specific phosphorylated peptides of interest are highlighted. Specifically STAT3 peptide containing Serine 727.

Previous work has shown pSTAT3 727 was involved in mitochondrial respiration (48, 49, 50), however, we did not see difference in growth rate (Supplemental Fig. 2*B*, 3, *H*–*K*) or apoptosis (Fig. 2, *D* and *E*, Supplemental Fig. 3, *F* and *G*). We next asked about STAT3 transcriptional targets. Interestingly, the decreased interaction of PD-L1 with pSTAT3 727 correlated with increased IL-6 production by the *Pdl1*^*CyMt*^ cells (Supplemental Fig. 5*C*), suggesting differences in regulation of pSTAT3 Tyr705 transcriptional targets. Finally, as differences in pERK, but not pP38, were demonstrated in dendritic cells in the *Pdl1*^*CyMt*^ mice after CCL21 stimulation (1), we next evaluated differences in either ERK or P38. We found no significant differences between *Pdl1* and *Pdl1*^*CyMt*^ in either ERK, pERK, P38, or pP38 levels after any of the indicated cytokine treatments (Supplemental Fig. 5, *D* and *E*). These data confirm that the TSS motif within PD-L1 cytoplasmic domain is required for interaction with the phosphorylated form, and more specifically serine 727, of STAT3, but not the native form of STAT3.

### PD-L1–paxillin interactions facilitate paxillin organization and cellular structure

**Figure 5:**
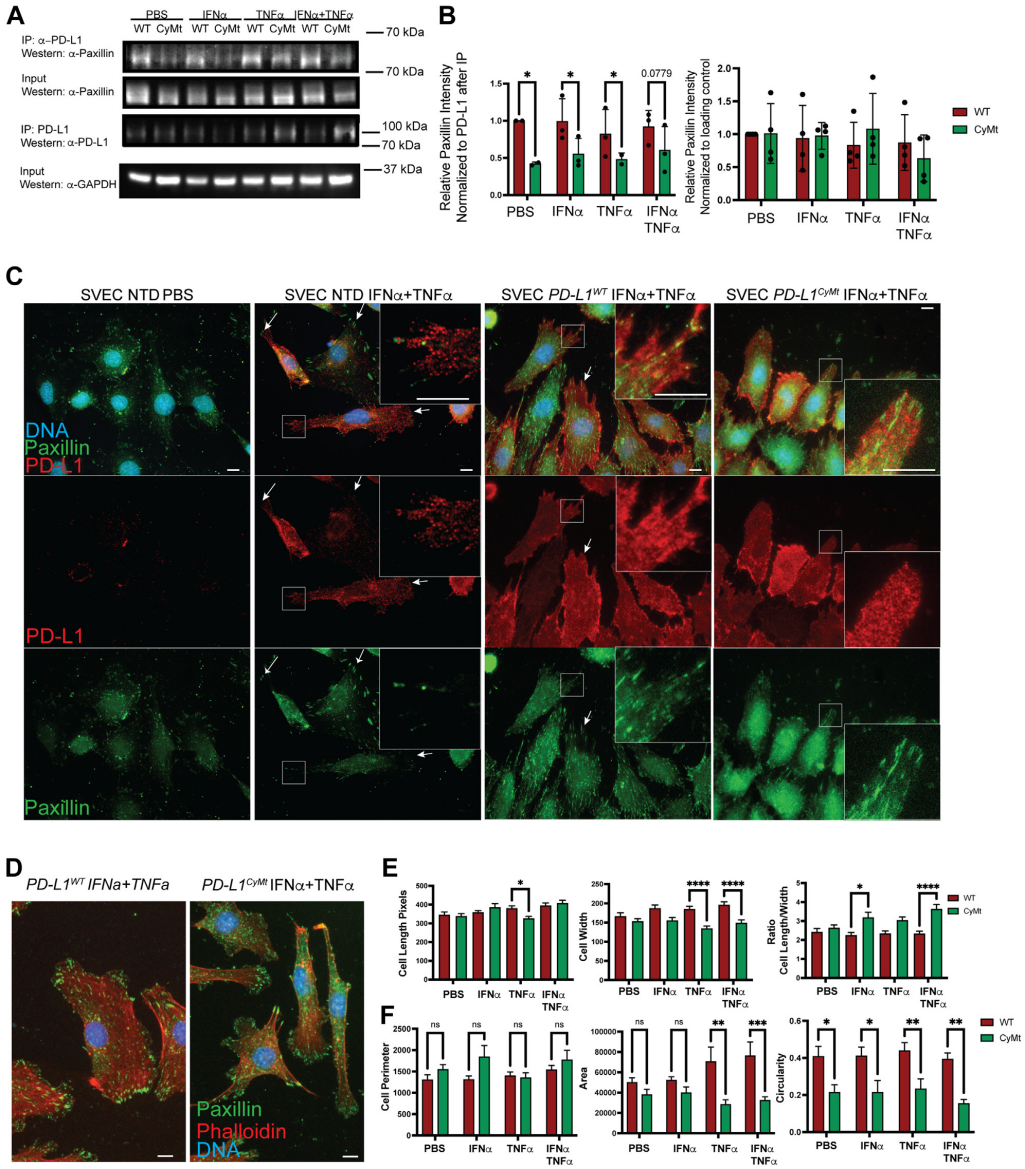
**Immunoprecipitation of PD-L1 after treatment with PBS, IFNα (500 U/ml), TNFα (100 ng/ml), or both for 30 min.***A*, western Blot probing for co-IP of Paxillin with WT *Pdl1* and *Pdl1*^*CyMt*.^ after stimulation. *B*, quantification of Paxillin coimmunoprecipitated with PD-L1, as well as in input, normalized to loading control. *C*, nontransduced SVEC cells in addition to WT *Pdl1* and *Pdl1*^*CyMt*^ were treated during a wound closure assay on gelatin-coated coverslips. Cells were stained for Paxillin and PD-L1. Inserts show PD-L1 organization at areas of paxillin marked focal adhesions. Arrows show other areas of PD-L1 and Paxillin colocalization. *D*, representative images showing Paxillin and F-actin representing defective cell spreading. *E* and *F*, quantification of cell morphological properties during wound closure assay (length, width, ratio, perimeter, area, circularity). Two-Way ANOVA performed on group analysis, for Western blot, quantification shows combined separate experiments, for cell morphology analysis, representative quantification shown for one of 3 independent experiments performed. The scale bar represents 10 μm for all images in (*C*) and (*D*). P = 0.10 to 0.05 *p*-value shown ∗=*p* < 0.05, ∗∗=*p* < 0.01, ∗∗∗=*p* < 0.001, ∗∗∗∗=*p* < 0.0001.

Paxillin is a focal adhesion protein that has been demonstrated to interact with pSTAT3 at the membrane to facilitate cell movement (34). Therefore, we next confirmed the MS data demonstrating a lack of interaction with Paxillin and PD-L1, when PD-L1 contained the CyMt mutation (Table 1, Fig. 5, Supplemental Fig. 4). Indeed, we found that Paxillin binding to PD-L1 was reduced in the *Pdl1*^*CyMt*^ mutant cells (Fig. 5*A*). The defect in actin polymerization we detected in cells (Fig. 3) and the disorganization of the lymphatics in the LN of *Pdl1*^*CyMt*^ mice (Fig. 1), suggested that these differences may be compounded by the inflammatory cytokines IFNα and TNFα. Therefore, we next asked if Paxillin interactions were impaired after treatment with either IFNα, TNFα, or both. We found that, indeed, after treatment of IFNα and TNFα that Paxillin bound to PD-L1 was still reduced in the *Pdl1*^*CyMt*^ cells, compared to WT, both following short (30 min) or overnight exposure to cytokines (Fig. 5, *A* and *B* and Supplemental Fig. 6, *A* and *B*) (1, 28). However, the diminished levels of paxillin pulled down with PD-L1 were not caused by changes in the level of total protein within the cells as Paxillin amounts were similar between WT *Pdl1* and *Pdl1*^*CyMt*^ cells (Fig. 5*A*). To determine if the differences in binding changed the localization of PD-L1 or Paxillin after treatment, we performed immunofluorescence in nontransduced (NTD) SVECs as well as WT *Pdl1* and *Pdl1*^*CyMt*^ transduced cells after IFNα and TNFα (Fig. 5*C*). We saw that in the NTD cells, as shown in Supplemental Fig. 2*E*, PD-L1 levels were minimal with no treatment, but following IFNα and TNFα, PD-L1 was upregulated and a portion of the endogenous PD-L1 localized to similar areas as paxillin (Fig. 5*C*). In the WT *Pdl1* transduced cells, PD-L1 and Paxillin localization was similar to the NTD cells (Fig. 5*C*). In the *Pdl1*^*CyMt*^, the Paxillin appeared localized to the cell body instead of at focal adhesions, and the PD-L1 appeared disorganized following IFNα and TNFα (Fig. 5*C*). We next asked if Paxillin could form proper focal adhesions connected to the actin cytoskeleton in the *Pdl1*^*CyMt*^ cells. Similar to Figure 3, Figure 5*C*, we found that in the *Pdl1*^*CyMt*^ cells Paxillin-mediated focal adhesions were disrupted and F-actin was disorganized during wound healing assay conditions (Fig. 5*D*). The *Pdl1*^*CyMt*^ expressing cells exhibited significant changes in morphology, a similar phenotype to either migrating adherent cells lacking STAT3 or defective cell spreading seen in paxillin-null cells (33, 51). To quantify this phenotype, we measured cell length, width, perimeter area, and circularity. We identified significant differences in circularity with or without cytokine treatment and significant differences in area, width, and length to width ratio only after cytokine treatment (Fig. 5, *D*–*F*). Taken together, these findings demonstrate that the TSS motif within the cytoplasmic tail of PD-L1 is necessary for cell morphology and cellular motility. These may be a result of the observed loss of interactions between PD-L1 and pSTAT3 and/or Paxillin required for proper focal adhesion formation.

## Discussion

In this article, we identify a mutation in PD-L1 that affects lymphatic organization in the LNs of mice injected with poly I:C. The flow cytometry data confirm that PD-L1 expression is dramatically upregulated following poly I:C on all LEC subsets within 24 h after injection, as we previously showed (11). Mutation of three residues in the cytoplasmic domain of PD-L1 (TSS-AAA) causes reorganization of the cortical/medullary LECs based on LN anatomy and results in fewer of the transcriptionally defined Ptx3/Marco LECs (MRC1+) (6). Unfortunately, it is difficult to distinguish the cortical LECs, defined anatomically, by their transcriptional profile. This may be due to the infrequency of cortical LECs in the LN or because the transcriptional profile is not different between medullary and cortical LECs (4, 6). Regardless, the differences we find *in vivo* in the different LEC subsets upon PD-L1 upregulation suggests PD-L1 has an important role in regulating LECs. We aimed to determine if PD-L1 upregulation caused by cytokines, such as type 1 IFN (11), defined the differences in the *Pdl1*^*CyMt*^ LECs or if the cytokines themselves were affecting the LECs independent of the coincident PD-L1 upregulation. To do this, we transduced either WT or mutant PD-L1 into the SVEC lymphatic cell line to induce constitutive expression that did not change with cytokine treatment (Supplemental Fig. 2*F*). Based on the data presented herein, it appears that it is not the induction of PD-L1 that is significant but instead the binding partners of PD-L1 that impart differences in the lymphatic endothelial cells upon cytokine exposure.

PD-L1 reverse signaling has been studied in multiple cell types and in each cell type PD-L1 has been shown to be involved in a variety of cell signaling pathways and mechanisms. Loss of PD-L1 reverse signaling in multiple cells types results in increased STAT3 Tyr705 phosphorylation. This increased Tyr705 phosphorylation is associated with caspase-mediated cell death in response to IFNβ in cancer cells (28); in T-cells, promotes Th17 responses (52); and has been reported in DCs (1), but the consequence of which is currently unknown. Many of these mechanisms only occur as a response to inflammatory cytokines such as type I and type II interferon, IL-6, TNFα, and those produced by TLR agonists (1, 11, 28, 52). These cytokines elicit a number of signaling pathways but overlap in STAT3 Tyr705 phosphorylation (53, 54), perhaps suggesting why loss of PD-L1 reverse signaling affects so many pathways and cell types in different ways. Here, we show that, in a cell line (SVEC) derived from murine LECs, that PD-L1 can form complexes with STAT3 and that the TSS domain of PD-L1 specifically affects the phosphorylation state of STAT3 both within and outside of this complex. STAT3, a transcription factor and protein involved in regulating cellular respiration and focal adhesions (33, 34, 40, 41, 42, 43, 44, 45, 46, 47, 55, 56) has multiple different impacts on cell phenotype. As we have shown PD-L1 interactions with STAT3, this may explain the multiple phenotypic outcomes seen in the absence of PD-L1 reverse signaling. One possible mechanism by which PD-L1 regulates STAT3 activity is *via* the regulation of the STAT3 phosphorylation state. Given our data, STAT3 pSer727 appears to be more readily and dynamically bound to PD-L1 compared to STAT3 pTyr705. STAT3 pSer727 is thought to be more important for cellular metabolism at the mitochondria (44, 45, 55), suggesting loss of STAT3 pSer727 interactions with PD-L1 could influence mitochondrial functions. Multiple studies have also demonstrated the STAT3 pSer727 can regulate STAT3 pTyr705 levels by destabilizing STAT3 homodimers and limiting transcription (46, 47). Our studies might suggest that increased pSer727 STAT3 bound to the PD-L1^CyMt^ is sequestering the STAT3 pSer727 and allowing for increased activation of STAT3 pTyr705. Loss of these interactions in the PD-L1^CyMt^ would thus increase pTyr705 STAT3 in favor of STAT3-mediated transcription and lead to increased IL6 production (Supplemental Fig. 4*C*). Our findings would suggest that PD-L1/pSTAT3/Paxillin complexes regulate focal adhesions and manipulation of these complexes could alter the balance of the different roles for STAT3. The exact mechanism of PD-L1 regulation of pSTAT3 is still not clear; however, these studies highlight the importance of PD-L1/STAT3 interactions to the cellular response to cytokines.

We also demonstrate the ability of PD-L1 to form complexes with Paxillin in addition to or together with pSTAT3. Another report has demonstrated the capacity of pSTAT3 to interact with Paxillin at focal adhesions, which promotes cellular movement (34). As our PD-L1 TSS-AAA mutation seems to both disrupt the amount of STAT3 phosphorylation as well as Paxillin levels bound to PD-L1, it seems likely that Paxillin and pSTAT3 interactions with PD-L1 are important for phospho-STAT3/Paxillin complex formation, which coordinates cellular movement. Indeed, we demonstrated altered SVEC cell movement and LN lymphatic vessel disorganization in the presence of inflammatory cytokines and that cellular morphology is significantly impaired. These data suggest that the mechanisms of PD-L1/STAT3/Paxillin to coordinate cell movement in the presence of inflammatory cytokines are important for *in vivo* immune responses. Indeed, in our previous paper (1), we demonstrated significant impairment of T-cell responses in our mouse model of *Pdl1*^*CyMt*^. While we attributed these differences to DCs, which also have defective actin polymerization and migration, it is now clear that loss of PD-L1 reverse signaling could also impact lymphatic reorganization, during an immune response, that may contribute to defective DC migration.

Several other potential PD-L1-binding partners are of particular interest based on studies of PD-L1 reverse signaling (1, 11, 25, 26, 29, 30, 31, 32, 52, 57, 58, 59). These include Arghap35, a Rho GTPase activating protein, and Ifih1, also known as MDA5, which is important for sensing dsRNA and affecting type 1 IFN responses (Table 2). Arhgap35 (Table 2), Arhgap29 (Table 1), and Arhgap5 (Supplemental Table 1) are of particular interest as Rho GTPases are well described to be involved in regulating actin where RhoA is important for protrusions of the lamellipodia (60, 61, 62), which are critical for cell migration. Other proteins were identified as bound to PD-L1 by MS that could be of consequence. One of which is ADP ribosylation factor4 (ARF4), a member of the ARF family proteins (Supplemental Table 1). ARF family proteins generally regulate endocytic vesicle trafficking from the golgi but downstream or alternative functions have been observed in migration, actin organization, and paxillin localization (63, 64, 65, 66). We also found additional ARF regulatory proteins bound to PD-L1 by MS, including, ADP-ribosylation factor GTPase-activating proteins 1, 2, 3 (ARFGAP1, ARFGAP2, ARFGAP3), ARF GTPase-activating protein GIT1 and GIT2 (GIT1, GIT2), Arf-GAP with coiled-coil, ANK repeat and PH domain-containing protein (ACAP2) Arf-GAP with Rho-GAP domain, ANK repeat and PH domain-containing protein 1 (ARAP1), and Brefeldin A-inhibited guanine nucleotide-exchange protein 2 (ARFGEF2) (Supplemental Table 1) (64, 67, 68, 69, 70, 71, 72). Intriguingly, GIT2 was demonstrated to bind to paxillin in another report (72). PIK3CB and PIK3C3, subunits of the PI3K, were also pulled out of the MS data (Supplemental Table 1). PI3K has been shown to be important for focal adhesions and cellular spreading in some cell types (73, 74, 75). MDA5 interaction with PD-L1 is also interesting as many of the differences we observe are following inflammatory cytokine (IFN alpha and TNF alpha) exposure that are regulated in part by the MDA5/RigI innate sensing pathway (54, 76, 77), which is critical for sensing dsRNA during the immune response. We do not yet understand how these other interactions are involved in regulating protection from cell death in cancer cells (28) and mice (32), promoting Th17 skewing (52) of T cells, or cellular migration of DCs (1) and LECs. Furthermore, it is possible that PD-L1 is part of a larger membrane-bound complex that contains multiple transmembrane or effector proteins.

As we begin to dissect the domains of PD-L1, the binding partners, and the functional consequences, use of this TSS-AAA mutation *in vitro* and *in vivo* will be important to delineate which alterations in the immune response are a consequence of PD-L1 forward and/or reverse signaling. Since PD-L1 is expressed by LECs, how alterations in reverse signaling may impact the immune response is critical as LECs utilize PD-L1 to promote peripheral tolerance *via* interaction with PD-1 on T cells (78, 79, 80, 81), as well as control LEC proliferation and viability during poly I:C injection (1, 11). These new findings, which demonstrate PD-L1 interactions can control cell movement, it will be important to understand how PD-L1 reverse *versus* forward signaling impacts the immune response.

## Experimental procedures

### Mice

Six- to eight-week-old male or female C57BL/6 or *Pdl1*^*CyMt*^ mice were used in experiments. No differences between male and female mice were detected. Mice were bred in-house or purchased through the NIH NCI at Charles River. All animal studies performed were approved by the Institutional Review Board and Institutional Animal Care and Use Committee at the University of Colorado Anschutz Medical Campus.

### LN dissection for flow cytometry

Mice were injected with poly I:C (5 μg/site) (Invitrogen) into both footpads and flank. Popliteal and inguinal LNs from mice after 24 h or naïve mice and LNs were digested as previously described (11). Once in single cell suspension, cells were stained with antibodies Caveolin-1 (1:200 Cell Signaling 3238S), ICAM-AF488 (1:600 Biolegend Clone YN1/1.7.4), Podoplanin-PE (1:200 Biolegend Clone 8.1.1), CD31 PerCP-cy5.5 (1:200 Biolegend Clone 390), CD206 PE-Cy7 (MRC-1) (1:100 Biolegend Clone C068C2), Lyve-1-APC (1:50 R&D Clone 223322), CD45-BV510 (1:300 Biolegend Clone 30-F11), and PD-L1-BV421 (1:200 Biolegend Clone 10F.9G2) 30 min at 4 °C and washed 2× with fluorescence activated cell sorting (FACS) (0.1% BSA, 0.02% sodium azide, 2mM EDTA in 1x Hank's Buffered Saline Solution pH 7.4). Samples were then stained with the secondary biotinylated anti-rabbit (1:100 Jackson Immunoresearch 111-066-003) 30 min at 4 °C, then washed 2x with FACS, then with the Streptavidin APC/Cy7 (1:200 Biolegend 405208) for 30 min at 4 °C, and washed 2× with FACS. For flow cytometry of SVECs, cells were treated with either PBS as mock, PBS, IFNα (Biolegend 752802), TNFα (Peprotech 315-01A-20 μg), or both IFNα and TNFα overnight. Cells were then trypsinized and mixed into a single cell suspension. Cells were then stained with PD-L1-PE (Biolegend 10F.9G2) for 30 min at 4 °C and analyzed by flow, similar to LN cells.

Samples were filtered and run on a BD LSR canto II flow cytometer using DIVA software (BD Biosciences) and analyzed with FlowJo software (Treestar).

### LN dissection and staining

Immunization and dissection performed similar to preparation for flow cytometry. LNs were removed and fixed in 16% buffered formalin phosphate and embedded in paraffin. Seven millimeter sections were cut and placed on slides. Prior to staining, slides were heated in 60 °C oven for 2 h to melt off paraffin wax. Slides were then rehydrated by washing with xylene twice for 10 min and then briefly washed in containers containing ethanol (EtOH) at 100%, 95%, 80%, and 75% before washing 3× briefly in deionized water. Slides then underwent antigen retrieval with antigen retrieval buffer pH 9 (AR900250ML- PerkinElmer) in pressure cooker on high for 15 min. Slides were then blocked with 5% normal donkey serum and 5% normal goat serum in 2.5% fetal bovine serum (FBS) in PBS (blocking buffer). Slides were then stained with hamster antipodoplanin (PDPN) (1:100 Biolegend Clone 8.1.1) and rabbit anti-Lyve-1-APC (R&D 223322 1:200) diluted in blocking buffer for 2 h room temperature (RT). Following primary antibody staining, slides were washed with 2.5% FBS in PBS 3 × 5 min and then stained in blocking buffer with a donkey antihamster Dylight 647 (1:500 Biolegend 405505). Slides were then washed and mounted in VectaShield mounting media containing 4′,6-diamidino-2-phenylindole (Vector Laboratories H-1200). Sections were imaged using a Nikon Eclipse Ti series fluorescent microscope. Images were taken with a Photometrics CoolSNAP DYNO fluorescent camera. For image quantification, regions of interest were drawn around the Lyve-1–positive regions to designate LECs within the either the cortical and medullary areas as defined by anatomical morphology.

### Cell lines (creation and culturing)

SVEC4-10 cells were purchased from (ATCC-CRL-2161). Cells were grown in Dulbecco’s modified Eagle’s medium (DMEM) high glucose (4.5 mg/ml) (Gibco) with 10% FBS (Atlanta biologicals S12450), as well as additives (1:100 each of: Hepes Corning 25-060-CI, sodium pyruvate Corning 25-000-CI, nonessential amino acids Corning 25-025-CI, L-glutamine Corning 25-005-CI, penicillin–streptomycin Sigma P4333-100ML), as well as 1.75 μl of 2-mercaptoethanol (Fisher Chemical BP176-100).

### Scratch assay

A 96-well ImageLock plate (Sartorius 4806) was coated with gelatin-based coating solution (Cell Biologics 6950) for 30 min at 37 °C. Cells were seeded onto gelatin-coated wells at 1.5e4 cells/well, grown to a confluent monolayer, in DMEM with 10% FBS, 4.5 g/ml glucose supplemented, then scratched using a 96-well Woundmaker (Sartorius). Following the scratch, media was immediately changed to serum-free media containing Incucyte Caspase-3/7 Red Dye for apoptosis (Sartorius 4707) with no supplements and either PBS, IFNα (Biolegend 752802), TNFα (Peprotech315-01A-20ug), or both IFNα and TNFα. Images were taken every 2 h with both phase and red on an Incucyte (Sartorius) to observe both wound closure and apoptosis. For immunofluorescent staining, cells were treated similarly after being grown on gelatin-coated glass coverslips in a 6-well plate. The coverslips were then scratched with a sterile pipette tip before fixation and staining at aforementioned indicated time points.

### Cytoskeletal G:F actin ratio

The F-actin/G-actin ratios were determined using Cytoskeleton kit (Cytoskeleton-BK037) using manufacturer’s protocol using secondary antibody antimouse IRDye 680 (LI-COR Biosciences-926-68070) diluted 1:20,000 for 1 h at RT. Membranes were then washed 3× and imaged on Bio-Rad ChemiDoc MP Imaging System.

### Immunofluorescence

About 22 mm × 22 mm glass coverslips (VWR-16004-302) were sterilized with 70% EtOH until use. Coverslips were then washed 1× with PBS before coating with collagen-based coating solution (Cell Biologics-6950) for 30 min at 37 °C in 6-well plates. Cells were then seeded at 0.3eˆ6 cells per well and grown on collagen-coated coverslips until 80% confluency. Cells were either scratched or left unscratched, then treated overnight with either PBS, IFNα (500U/ml), TNFα (100 ng/ml), or both overnight. Then, coverslips were stained. For F-actin, Cytoskeleton F-actin Visualization kit was used (Cytoskeleton-BK005) with or without Paxillin (Thermo Scientific Clone 5H11 1:50) with secondary goat-antimouse AF633 (1:500 Invitrogen A21126) following Cytoskeleton manufacturer’s instructions. Five percent goat serum in 2.5% FBS/PBS was used as blocking buffer. For PD-L1 staining, cells were stained with PD-L1-PE (1:200 BioLegend clone 10F.9G2) with 24G2 block prior to fixation. Cells were then washed and fixed/stained for paxillin similar to the Cytoskeleton kit’s instructions (4% paraformaldehyde, 3% TritonX-100 in PBS used as fix and perm buffers). Cells were visualized on the Nikon eclipse Ti Series fluorescent microscope and images were captured using the Photometrics CoolSNAP DYNO. Cell measurements were taken in Adobe Photoshop using the measure tool and object selection tool.

### Live cell F-actin microscopy

Cells were grown in 6-well plates to confluence. Then, cell monolayer was scratched with a sterile P1000 pipette tip. Media was immediately changed to treatment (similar to scratch assay treatment) that additionally contained the live cell F-actin probe (SiR-actin) from Cytoskeleton (CY-SC006) at 100 nM (1:10,000). Next day, cells were imaged on Olympus IX83 live cell apparatus, using the 10× magnification for plastic tissue culture plates. Individual 10× images were captured in the Cy5 channel every 10 min to visualize F-actin reorganization and cellular movement across the scratch.

### Immunoprecipitation

Cells were grown to 90% confluency, then treated overnight with either sodium fluoride (1 mM), sodium vanadate (0.5 mM), TNFα (100 ng/ml), and IFNα (500U/ml) overnight. Cells were lysed using lysis buffer comprised of 90% m-PER (Thermo Scientific 78503) with the added sodium fluoride (50 mM Final) sodium vanadate (1 mM Final), and Complete Protease Inhibitor cocktail (1× Final Sigma-Aldrich 11873580001). Cells were lysed for 10 min on ice with vortexing every 5 min. Then, lysate was clarified with a 15 min spin at 20,000×*g*. Lysate protein concentration was determined with bicinchoninic acid, and equivalent protein amounts were added to new tubes and diluted to equivalent concentrations with lysis buffer. Glutathione-*S*-transferase (GST)-tagged GFP nanobody was made in house using vector pGEX6P1-GFP-Nanobody, which was a gift from Kazuhisa Nakayama (Addgene plasmid #61838; http://n2t.net/addgene:61838; RRID: Addgene_61838). GST beads for immunoprecipitation were purchased from GoldBio (Cat No. G-250-5). Seventy-five microliters of 50% beads/PBS were isolated per approximately 1 mg of cell lysate.

Sample was incubated overnight on rotator with 75 μl of 50% beads/PBS per 1 mg of cell lysate with 10 μl of GST-tagged anti-GFP nanobody (5 mg/ml). Beads were then washed 4× in Tris-buffered saline with 0.1% Tween-20. Sample was eluted by adding 25 μl of 4× Laemmli with 10% 2-mercaptoethanol, followed by heating in heat block ∼90 °C for 10 min. Sample was then either analyzed by mass spectrometry as described or by Western blot, by running sample on Tris-glycine acrylamide gels (10%).

### Mass spectrometry

Global bottom-up LC-MS/MS analysis.

#### Experimental design and rationale

All samples were processed in a blinded fashion and no data points were excluded. N = 6 samples per cell type were loaded onto a 1.5 mm thick NuPAGE Bis-Tris 4% to 12% gradient gel (Invitrogen). The BenchMark Protein Ladder (Invitrogen) was used as a protein molecular mass marker. The electrophoretic run was performed by using Mes SDS running buffer in an X-Cell II mini gel system (Invitrogen) at 200 V, 120 mA, 25 W per gel for 30 min. The gel was stained using SimplyBlue SafeStain (Invitrogen) stain and destained with water according to the manufacturer’s protocol. Each lane of the gel was divided into four equal-sized bands, and proteins in the gel were digested as follows. Gel pieces were destained in 200 μl of 25 mM ammonium bicarbonate in 50 % v/v acetonitrile for 15 min and washed with 200 μl of 50% (v/v) acetonitrile. Disulfide bonds in proteins were reduced by incubation in 10 mM DTT at 60 °C for 30 min and cysteine residues were alkylated with 20 mM iodoacetamide in the dark at RT for 45 min. Gel pieces were subsequently washed with 100 μl of distilled water followed by addition of 100 ml of acetonitrile and dried on SpeedVac (Savant ThermoFisher). Hundred nanograms of trypsin was added to each sample and allowed to rehydrate the gel plugs at 4 °C for 45 min and then incubated at 37 °C overnight. The tryptic mixtures were acidified with formic acid up to a final concentration of 1%. Peptides were extracted two times from the gel plugs using 1% formic acid in 50% acetonitrile. The collected extractions were pooled with the initial digestion supernatant and dried on SpeedVac (Savant ThermoFisher). Samples were desalted on Thermo Scientific Pierce C18 Tip.

#### Phosphopeptide enrichment

Phosphopeptide enrichment was performed on n = 3 samples per group using Hight-Select Fe-NTA Phosphopeptide Enrichment Kit according to the manufacturer’s instructions and supplied buffers. The dry phosphopeptides were resuspended in 200 μl of binding/wash buffer and incubated with Fe–nitrilotriacetic acid beads for 30 min at RT. Three 200 μl washes with binding/wash buffer were performed. Phosphopeptides bound to the Fe–nitrilotriacetic acid beads were eluted twice with 100 μl of elution buffer. The eluent was dry immediately in a SpeedVac concentrator.

#### Analysis of peptides

A 20 μl of each sample was loaded onto individual Evotips for desalting and then washed with 20 μl 0.1% formic acid, followed by the addition of 100 μl storage solvent (0.1% formic acid) to keep the Evotips wet until analysis. The Evosep One system (Evosep) was used to separate peptides on a Pepsep column (150 um inter diameter, 15 cm) packed with ReproSil C18 1.9 um, 120A resin. The system was coupled to the timsTOF Pro mass spectrometer (Bruker Daltonics) *via* the nanoelectrospray ion source (Captive Spray, Bruker Daltonics). The mass spectrometer was operated in PASEF mode. The ramp time was set to 100 ms and 10 PASEF MS/MS scans per topN acquisition cycle were acquired. MS and MS/MS spectra were recorded from m/z 100 to 1700. The ion mobility was scanned from 0.7 to 1.50 Vs/cm^2^. Precursors for data-dependent acquisition were isolated within ± 1 Th and fragmented with an ion mobility–dependent collision energy, which was linearly increased from 20 to 59 eV in positive mode. Low-abundance precursor ions with an intensity above a threshold of 500 counts but below a target value of 20,000 counts were repeatedly scheduled and otherwise dynamically excluded for 0.4 min.

#### Database searching and protein identification

MS/MS spectra were extracted from raw data files and converted into .mgf files using MS Convert (ProteoWizard, Ver. 3.0). Peptide spectral matching was performed with Mascot (Ver. 2.5) against the Uniprot mouse database. Mass tolerances were ± 15 ppm for parent ions and ± 0.4 Da for fragment ions. Trypsin specificity was used, allowing for one missed cleavage. Met oxidation, protein N-terminal acetylation, peptide N-terminal pyroglutamic acid formation, and Phospho (STY) were set as variable modifications with Cys carbamidomethylation set as a fixed modification.

Scaffold (version 4.9, Proteome Software) was used to validate MS/MS-based peptide and protein identifications. Peptide identifications were accepted if they could be established at greater than 95.0% probability as specified by the Peptide Prophet algorithm. Protein identifications were accepted if they could be established at greater than 99.0% probability and contained at least two identified unique peptides.

### Statistical analysis

Data were analyzed using Prism9 (GraphPad). Data were either analyzed by *t* test or one-way ANOVA when multiple comparisons were required. Wound closure assays were analyzed by generating linear best-fit lines and determining *p*-value for differences in slope. Each experiment was performed with three to seven replicates and at least two to three times with similar results.

## Data availability

All data are contained within the article except Supplemental Table 1 which is mass spectrometry data. Mass spectrometry data have been deposited at Center for Computation Mass Spectrometry (CCMS). Follow the instructions contained within the url to view the data: https://massive.ucsd.edu/ProteoSAFe/private-dataset.jsp?task=f20bd28b30434e42ae527a0223f08c9e.

## Supporting information

This article contains supporting information (Supplemental Figs. 1–6, Supplemental Table 1 and Movies 1–8).

## Conflict of interest

The authors declare that they have no conflicts of interest with the contents of this article.
