# Supplementary material for: Programmed death ligand 1 intracellular interactions with STAT3 and focal adhesion protein Paxillin facilitate lymphatic endothelial cell remodeling
Source: J Biol Chem. 2022 Nov 12;298(12):102694. doi: 10.1016/j.jbc.2022.102694 (PMC9761386; doi:10.1016/j.jbc.2022.102694)
Supplement: Supplementary Figures [file mmc9.docx]

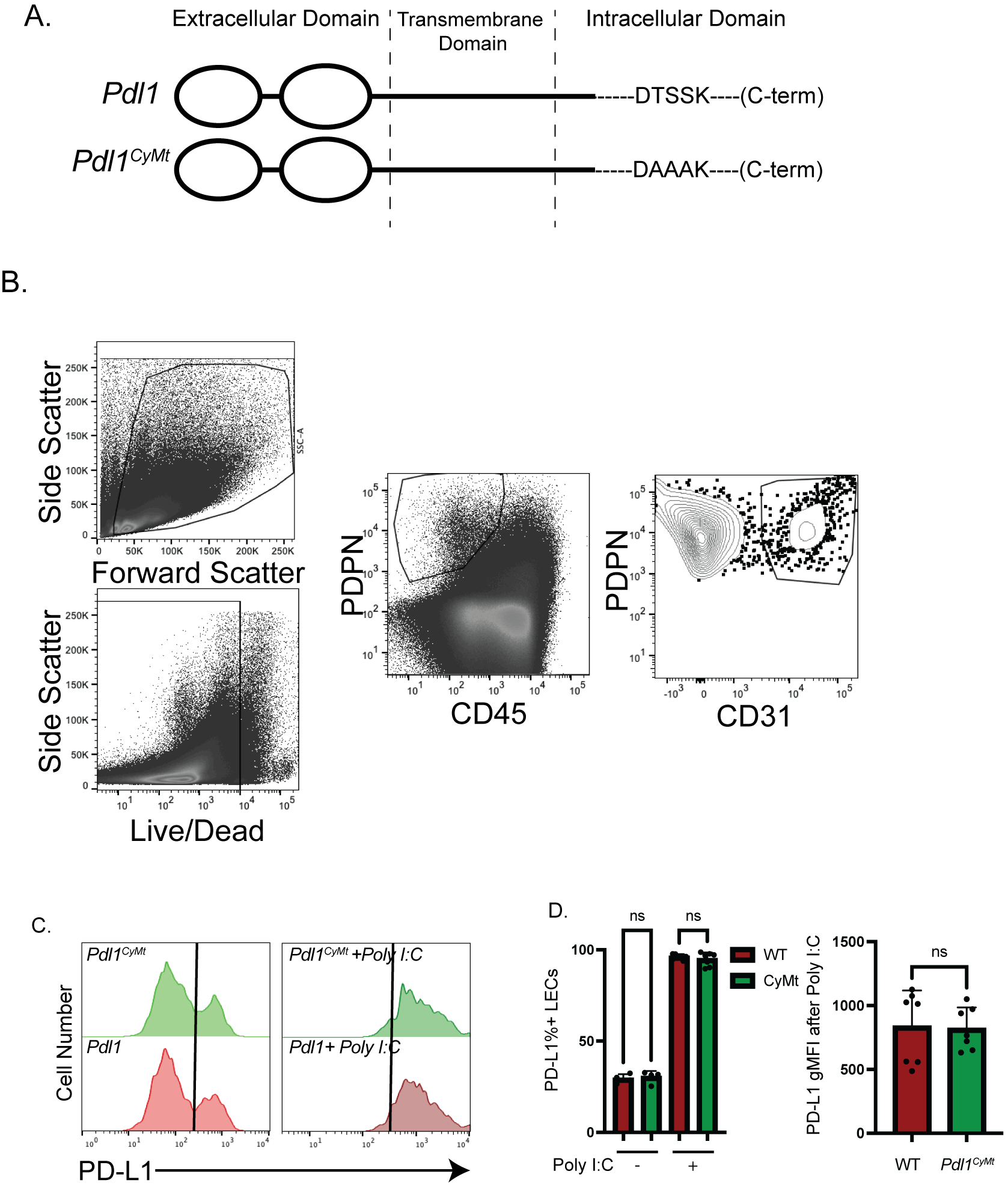


Supplemental Figure 1. LECs from WT and *Pdl1^CyMt^* mice have similar expression of PD-L1 following Poly I:C. A. Schematic showing mutation in cytoplasmic domain of PD-L1 in WT *Pdl1* compared to *Pdl1^CyMt^* mice. B. Gating strategy to analyze total LEC population. C,D. LN cells isolated from popliteal LNs from either naïve or 24 hours after polyI:C injection. Surface expression of PD-L1 was analyzed. Shown is percent positive for PD-L1 as well as geometric mean fluorescence intensity (gMFI) on LECs. LNs were analyzed from 3 experiments with 2-3 mice per group.

_
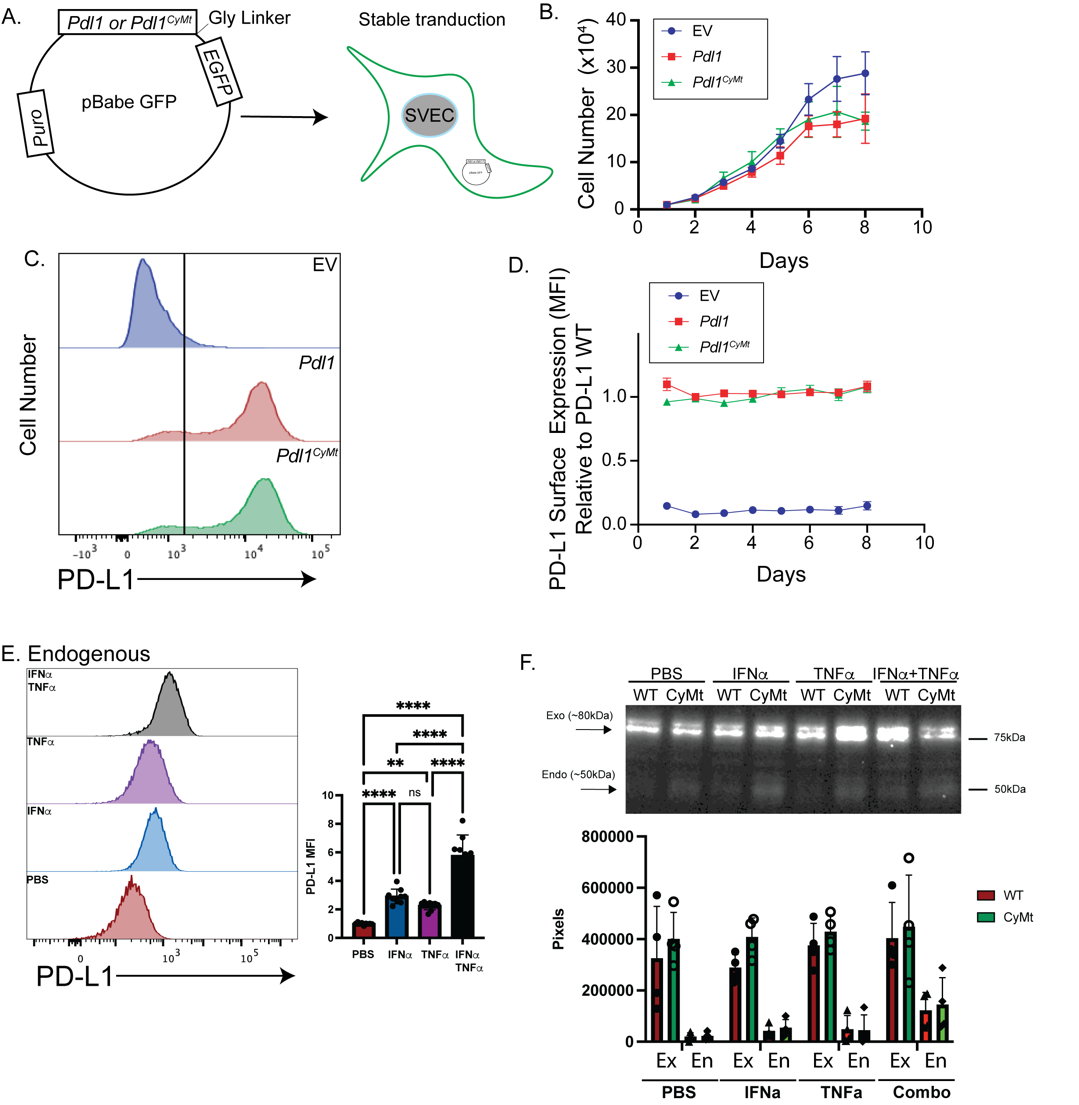
_

Supplemental Figure 2. Transduction of WT *Pdl1* or *Pdl1^CyMt^* does not affect growth rate or PD-L1 surface expression. A. SVEC 4-10 cells were transduced with lentiviral pBABE-GFP expression vectors containing either no insert (EV), WT *Pdl1* or *Pdl1^CyMt^*. Following selection with puromycin transduced cells were seeded into wells of 24-well plates at 1.5e^4^ cells per well. Three wells were collected each day per genotype and counted to determine growth rate over the indicated time. Shown are compiled results from three independent experiments. C. Cells at each timepoint, from the time course in B, were stained with anti-PD-L1 and levels were measured on a flow cytometer. D. Quantification of mean fluorescence intensity of surface PD-L1 on SVEC4-10 cells with indicated expression vector. E. Non-transduced SVEC4-10 cells (SVEC NTD) were treated with IFNα (500U/mL), TNFα(100ng/mL), or both IFNα and TNFα. Cells were then stained for surface PD-L1 and analyzed by flow cytometry. F. PD-L1 was analyzed by western blot in transduced cells to show endogenous PD-L1 (~50kDa) levels compared to GFP tagged PD-L1 constructs (~80kDa). For growth rate and PD-L1 expression, three independent experiments with at least three replicates per group were assayed and analyzed Line of best fit test showed no significant difference between WT *Pdl1* and *Pdl1^CyMt^* expressing Cells. SVEC NTD cell treatments were repeated 3 times with 3 wells per experiment. All data is shown, normalized to mean PBS MFI for each repeat. PD-L1 analysis by western blot is shown for 4 independent experiments. Data was analyzed using Prism two-way ANOVA **=p<.01, ****=p<.0001


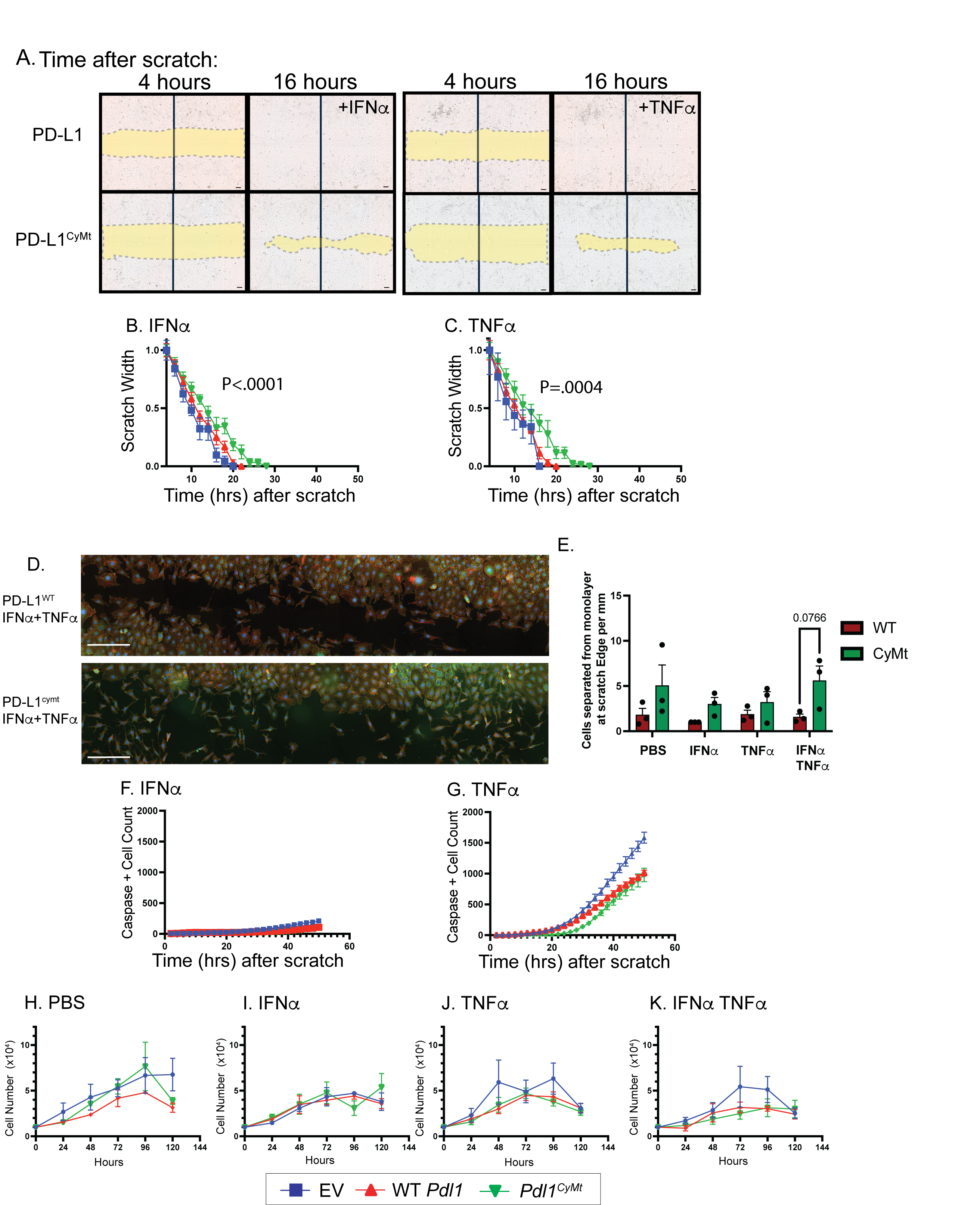


Supplemental Figure 3. SVEC 4-10 Cells containing EV, *Pdl1* or *Pdl1^CyMt^* vectors were plated in 5-wells per group per treatment, of an Image-Lock 96-well plate, at 1.5e^4^ cells/well. Cells were allowed to grow to confluence ~36 hours. Confluent cells were scratched with a Sartorius Woundmaker. Immediately after scratch, media was changed to Serum free MEM media containing either TNFα (100ng/ml) or IFNα (500U/ml). Cells were imaged every 2 hours using an IncuCyte. A. Representative images and representative graphs are shown from three independent experiments, 5-7 wells each. B,C. Quantification of scratch width over time after treatment. Graph is of representative experiment. Assay was repeated 3 independent times with similar results. P-value for difference in slope of WT *Pdl1* vs. *Pdl1^CyMt^* best fit line shown. D,E Migration pattern during wound closure appeared different between WT *Pdl1* and *Pdl1^CyMt^* expressing cells, representative images shown and quantification of individual cells migrating ahead of wound edge shown. For visualization, Phalloidin stained F-actin is in red, Paxillin in Green and DAPI in blue. F,G. Number of Active Caspase 3 reagent positive cells to determine the number of apoptotic cells per image over time. No significant differences were found in caspase 3 between WT *Pdl1*and *Pdl1^CyMt^*. H-K Growth curve was performed while undergoing treatment in serum free minimal essential media. Line of best fit analysis showed no significant difference between WT *Pdl1* and *Pdl1^CyMt^*. For scratch assay, representative experiment is shown of three independent experiments. For wound closure differences, and growth curves combined data from 3 independent experiments is shown. For p<.01, p-value is labeled. Scale Bar in A is 100μm. Scale bar in D is 250 μm.


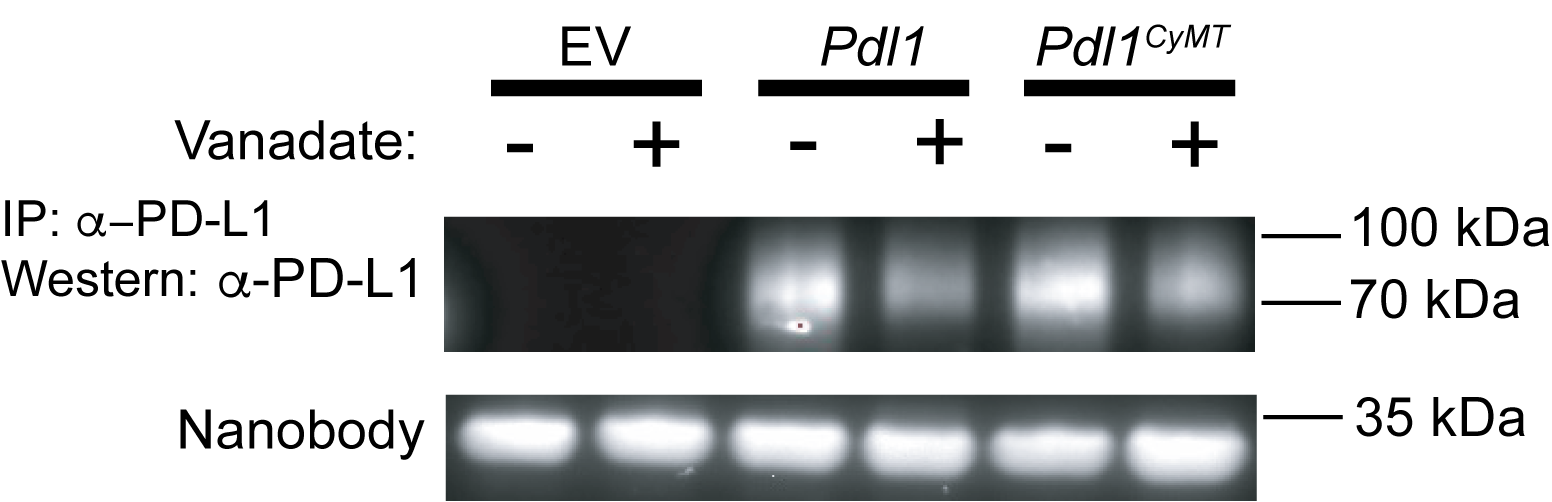


Supplemental Figure 4. SVEC 4-10 Cells expressing GFP, *WT Pdl1*-GFP, or *Pdl1^CyMt^*-GFP were grown in 2 10cm dishes and treated with PBS of Vanadate phosphatase inhibitor, then lysed in MPER buffer. Then GFP was immunoprecipitated. These samples were sent for Mass-Spec Analysis. A Small fraction of IP was analyzed by western blot to confirm similar levels of PD-L1 construct was pulled down between WT *Pdl1* and *Pdl1^CyMt^* samples. Anti-GFP nanobody band is shown to demonstrate equivalent levels of nanobody used to pull down PD-L1.


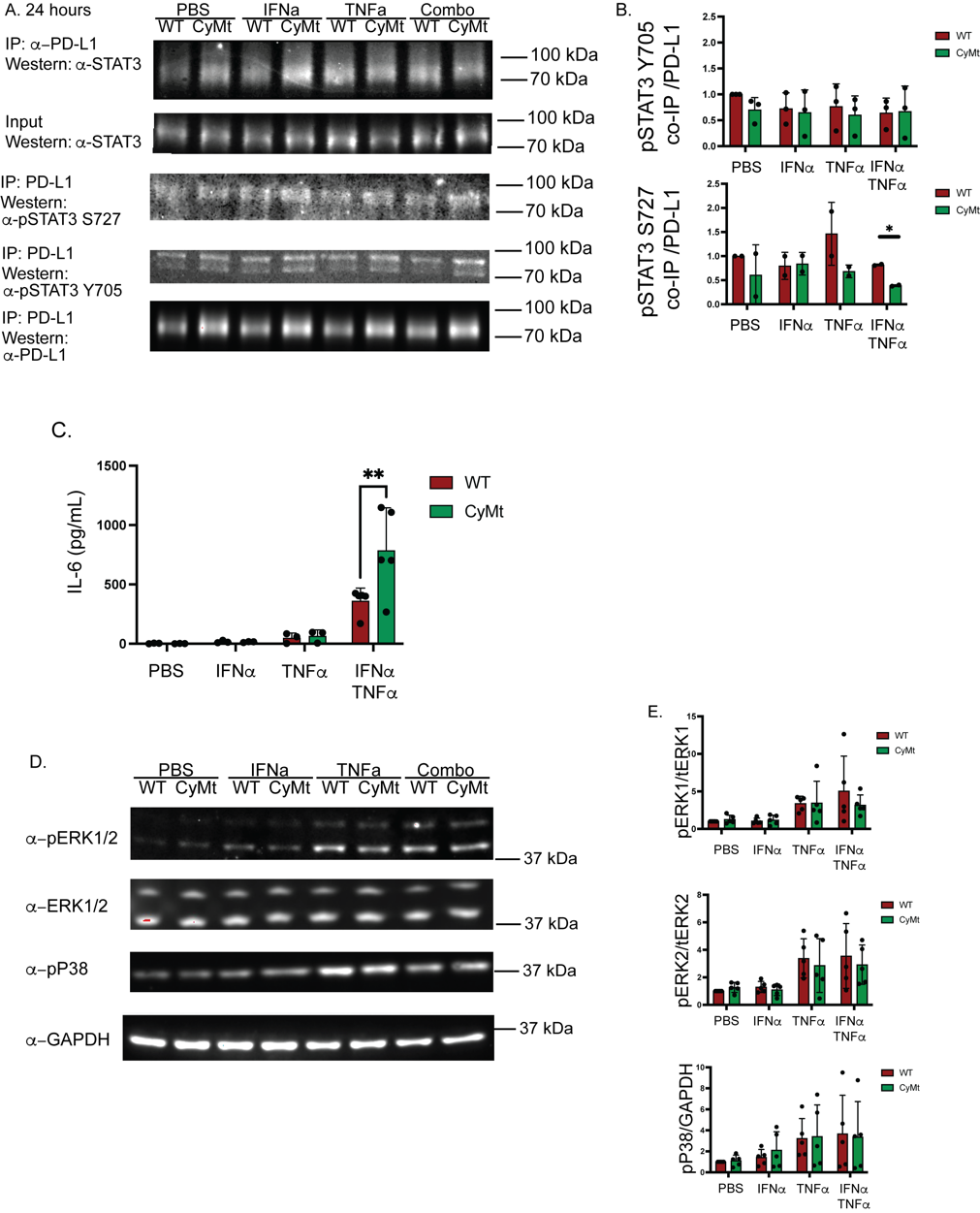


Supplemental Figure 5. A. SVEC 4-10 Cells containing WT *Pdl1-GFP* or *Pdl1^CyMt^-GFP* vectors were plated in 10 cm dishes. Once at 90% confluency, media was changed to serum free MEM media containing either PBS, TNFα (100ng/ml), IFNα (500U/ml), or both TNFα (100ng/ml) and IFNα (500U/ml). The following day cells were lysed and PD-L1-GFP was immunoprecipitated. A,B. Western Blot Analysis of pSTAT3 pulled-down with PD-L1 following overnight stimulation with IFNα and TNFα or Both. Experiments were excluded if signal could not be detected above background. Input for pSTAT3 on both sites was below limit of detection. C. Media was analyzed by ELISA following stimulation overnight to determine concentrations of IL-6 in Media secreted by cells. D E. Input was analyzed for changes in signaling proteins pERK1/2 and pP38. Quantification shows combined independent experiments normalized to PBS treated WT *Pdl1*. Data was analyzed using Prism two-way ANOVA *=p<.05, **=p<.01


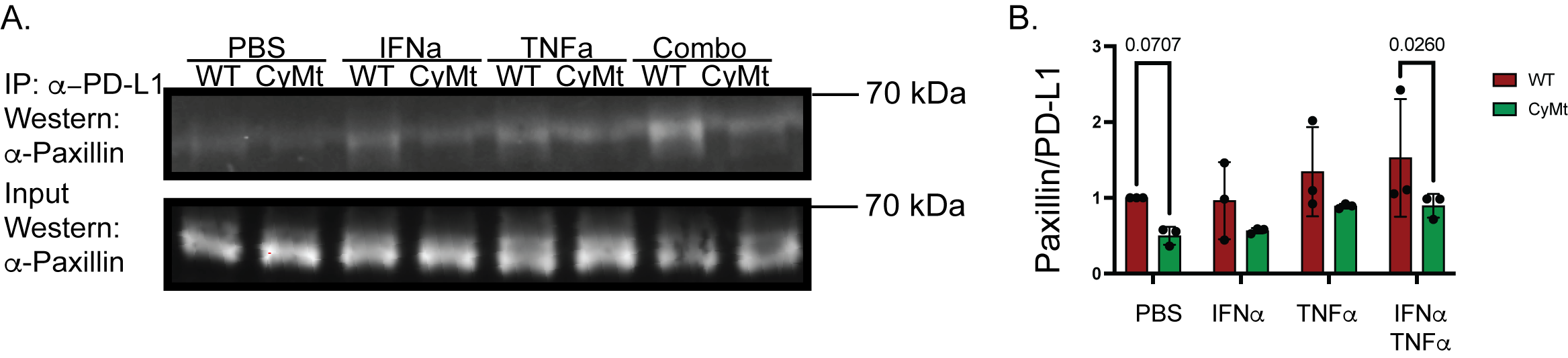


Supplemental Figure 6. Immunoprecipitation of PD-L1 after treatment with PBS, IFNα (500U/mL), TNFα (100ng/mL), or both IFNα and TNFα overnight. A. Western Blot probing for co-IP of Paxillin with WT *Pdl1* and *Pdl1^CyMt^* after indicated stimulation. B. Quantification of Paxillin coimmunoprecipitated with PD-L1. Western blot was analyzed using Prism 2-way ANOVA Pairing matched batches of three combined independent experiments. p-values shown for those less than 0.10
